# Giant left atrial thrombi despite surgical left atrial appendage resection in rheumatic heart disease: a case report

**DOI:** 10.1093/ehjcr/ytag778

**Published:** 2026-10-26

**Authors:** Takashi Akima, Katsutoshi Sekine, Tomoaki Hirasawa, Yuki Endo

**Affiliations:** Department of Internal Medicine, Saitama City Hospital, 2460 Mimuro, Midori-ku, Saitama 336-8522, Japan; Department of Internal Medicine, Saitama City Hospital, 2460 Mimuro, Midori-ku, Saitama 336-8522, Japan; Department of Internal Medicine, Saitama City Hospital, 2460 Mimuro, Midori-ku, Saitama 336-8522, Japan; Department of Cardiovascular Surgery, Saitama City Hospital, 2460 Mimuro, Midori-ku, Saitama 336-8522, Japan

**Keywords:** Left atrial thrombus, Bioprosthetic mitral valve, Rheumatic heart disease, Atrial fibrillation, Case report

## Abstract

**Background:**

Although surgical left atrial appendage resection reduces a major source of thromboembolism, left atrial cavity thrombosis may still occur in patients with persistent severe atrial stasis.

**Case summary:**

An 86-year-old woman was admitted with severe acute decompensated heart failure and a low-output state. She had long-standing persistent atrial fibrillation and had undergone bioprosthetic mitral valve replacement with surgical left atrial appendage resection for rheumatic mitral stenosis one year earlier. She received warfarin for the first 3 months after surgery, followed by edoxaban 30 mg once daily; medication reconciliation on admission suggested suboptimal adherence. Contrast-enhanced computed tomography on Day 2 showed two giant left atrial filling defects. Pretreatment laboratory findings yielded an International Society on Thrombosis and Haemostasis overt disseminated intravascular coagulation score of 6; antithrombin activity was 69.4%, and no overt bleeding was present. Unfractionated heparin (UFH) and heart failure therapy were initiated; fresh-frozen plasma and antithrombin concentrate were administered. Coagulation variables improved, and the filling defects disappeared by Day 39 during ongoing UFH and heart failure therapy, without systemic embolism. After stabilization, warfarin therapy was reintroduced with adherence support.

**Discussion:**

In this case, severe rheumatic atrial remodelling, marked left atrial enlargement, persistent atrial fibrillation, impaired forward flow, and uncertain anticoagulant exposure probably contributed to giant left atrial cavity thrombus formation despite prior appendage resection. This case highlights that surgical left atrial appendage resection does not eliminate the risk of left atrial cavity thrombosis when severe atrial stasis persists.

Learning points

- Surgical left atrial appendage resection does not eliminate the risk of left atrial cavity thrombosis when severe atrial stasis persists.
- When thrombosis occurs during prescribed anticoagulation, dose eligibility and adherence should be assessed before concluding drug failure.

## Introduction

The left atrial appendage is the usual site of thrombus formation in atrial fibrillation, but thrombi may also form elsewhere in the left atrial cavity when marked remodelling, persistent atrial fibrillation, and severe stasis coexist.^1–5^ Anticoagulation is complex in rheumatic heart disease after bioprosthetic mitral valve replacement: Vitamin K antagonists remain strongly supported for rheumatic atrial fibrillation, whereas direct oral anticoagulants (DOACs) may be considered in selected patients with bioprosthetic valves.^1,2,6–10^ We report giant left atrial cavity thrombi after surgical appendage resection, accompanied by consumptive coagulopathy compatible with overt disseminated intravascular coagulation.

## Summary figure

**Figure:**
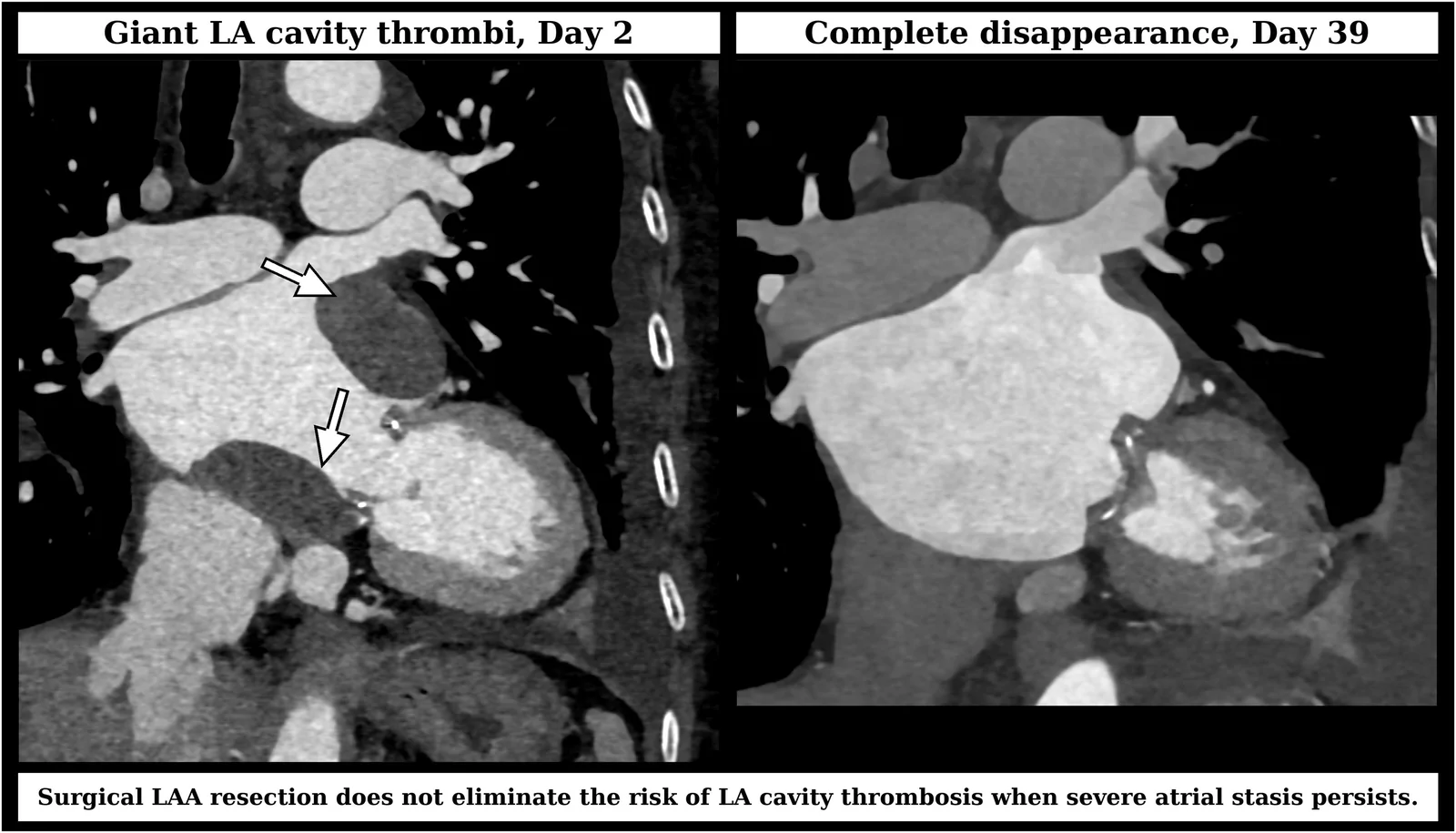
Contrast-enhanced computed tomography on Day 2 showed giant left atrial cavity thrombi after surgical left atrial appendage resection, in the setting of rheumatic atrial remodelling, long-standing persistent atrial fibrillation, severe atrial stasis, and uncertain anticoagulant exposure. The thrombi had completely disappeared by Day 39 during continuous intravenous unfractionated heparin and heart failure therapy, without systemic embolism. LA, left atrium; LAA, left atrial appendage.

## Case presentation

An 86-year-old woman was admitted with progressive dyspnoea and hypoxaemia in the setting of acute decompensated heart failure and a low-output state. She had long-standing persistent atrial fibrillation and had undergone bioprosthetic mitral valve replacement with surgical left atrial appendage resection for rheumatic mitral stenosis one year earlier. After 3 months of postoperative warfarin, the prescribed pre-admission edoxaban dose was 30 mg once daily. Medication reconciliation suggested suboptimal adherence, although the number of missed doses was unknown.

On admission, blood pressure was 117/93 mmHg, heart rate 146 beats/min in atrial fibrillation, and respiratory rate 24 breaths/min. Chest radiography showed cardiomegaly, pulmonary congestion, and pleural effusions (*Figure 1E*). The platelet count was 6.5 × 10^4^/μL (reference range, 15.8–34.8 × 10^4^/μL) on Day 1. Pretreatment values on Day 2 included platelet count 6.7 × 10^4^/μL, fibrinogen 58 mg/dL (181–378 mg/dL), fibrin/fibrinogen degradation products (FDP) 88.7 μg/mL (≤5.0 μg/mL), D-dimer 11.0 μg/mL (<1.0 μg/mL), prothrombin time (PT) 16.4 s (10.5–12.2 s), and antithrombin activity 69.4% (80.0–130%). The International Society on Thrombosis and Haemostasis (ISTH) overt disseminated intravascular coagulation (DIC) score was 6, compatible with overt DIC; no overt bleeding was present.^11^ Serial laboratory results and institutional reference ranges are provided in *Table 1*.

**Figure 1:**
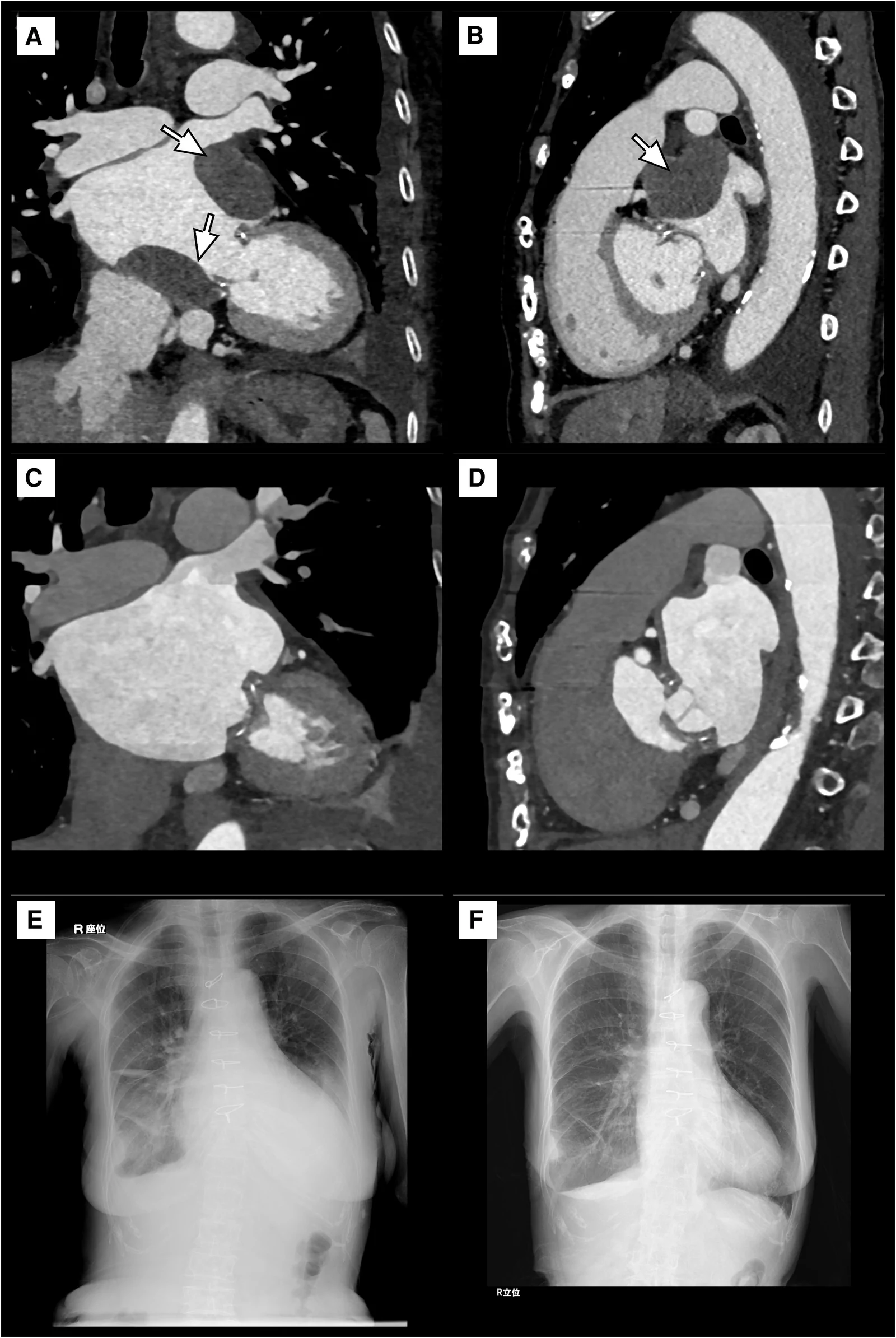
Serial imaging findings. Contrast-enhanced computed tomography on Day 2 showed two giant filling defects in the left atrial cavity (*A*, coronal view; B, sagittal view; arrows). Repeat contrast-enhanced computed tomography on Day 39 showed complete disappearance of the thrombi (*C*, coronal view; D, sagittal view). Chest radiographs obtained on admission (*E*) and after heart failure treatment on Day 57 (*F*) showed improvement in pulmonary congestion and pleural effusions.

**Table 1:** Serial changes in coagulation and renal function parameters and coagulation-related treatments

Parameter	Reference range	Day 1	Day 2	Day 3	Day 7	Day 10	Day 14
Platelet count, ×10^4^/μL	15.8–34.8	6.5	6.7	7.1	13.0	12.0	14.3
Fibrinogen, mg/dL	181–378		58	105	270	214	267
FDP, μg/mL	≤5.0		88.7	12.8	4.0	7.1	5.9
D-dimer, μg/mL	<1.0	9.9	11.0	3.7	2.2	5.1	4.3
PT, s	10.5–12.2	15.7	16.4		12.0	11.8	12.3
PT-INR	0.91–1.08	1.46	1.53		1.10	1.06	1.13
Antithrombin activity, %	80.0–130		69.4	80.6			87.1
APTT, s	24–34	34.5	39.1	50.5	44.6	54.2	61.9
Serum creatinine, mg/dL	0.46–0.79	1.67	1.53	1.21	1.28	1.01	1.06
eGFR, mL/min/1.73 m^2^	—	23.2	25.5	33.0	31.0	40.2	38.1
Unfractionated heparin infusion	—		Started	Continued			
Fresh-frozen plasma, mL/day	—		480	960			
Antithrombin concentrate, IU/day	—		1000				

Day 2 values preceded UFH, FFP, and antithrombin administration. The ISTH overt DIC score was 6 (platelet count, 1 point; FDP, 3; PT prolongation, 1; fibrinogen, 1); institutional reference intervals were 10.5–12.2 s for PT and ≤5.0 μg/mL for FDP. Day 3 values were obtained 17 h after Day 2 FFP 480 mL and antithrombin concentrate 1000 IU, during ongoing UFH and before Day 3 FFP 960 mL. UFH targeted an APTT 1.5–2.0 times the control value and continued through Day 39. Blank cells indicate that a parameter was not measured or a treatment was not administered on that day, as applicable. Reference ranges are those of the treating institution; the creatinine reference range is for women. A dash (—) indicates that no reference interval is specified for eGFR or that a reference range is not applicable to a treatment. Laboratory reference ranges should be distinguished from anticoagulation treatment targets.

APTT, activated partial thromboplastin time; DIC, disseminated intravascular coagulation; eGFR, estimated glomerular filtration rate; FDP, fibrin/fibrinogen degradation products; FFP, fresh-frozen plasma; INR, international normalized ratio; ISTH, International Society on Thrombosis and Haemostasis; IU, international unit; PT, prothrombin time; UFH, unfractionated heparin.

Transthoracic echocardiography showed severe left atrial enlargement (diameter 42 mm; volume index 78 mL/m^2^), a severely reduced left ventricular ejection fraction of 13%, and a markedly reduced left ventricular outflow tract velocity–time integral of less than 5 cm. The bioprosthetic mitral valve showed no evidence of an abnormal transprosthetic gradient (mean gradient, 4 mmHg). Non-contrast computed tomography (CT) was non-diagnostic. Contrast-enhanced CT on Day 2 showed two low-attenuation left atrial filling defects measuring 51 × 22 × 34 mm and 43 × 24 × 43 mm (*Figure 1A* and *B*, white arrows). Apical four- and three-chamber views demonstrated round, hyperechoic left atrial masses with apparently homogeneous internal echotexture adjacent to the mitral valve and annulus (see Supplementary material online, *Videos S1* and *S2*).

Continuous intravenous unfractionated heparin (UFH) was started, targeting an activated partial thromboplastin time (APTT) 1.5–2.0 times the control value. Because of profound hypofibrinogenaemia, fresh-frozen plasma (FFP) 480 mL was administered on Day 2 and 960 mL on Day 3. A single 1000-unit dose of antithrombin concentrate was given on Day 2 because antithrombin activity was reduced. Oxygen, diuretics, and low-dose dobutamine at 3 μg/kg/min were also provided. Surgical thrombectomy was considered excessively risky because of advanced age, frailty, previous cardiac surgery, and clinical instability. Because the large thrombus burden was considered potentially haemodynamically relevant, therapeutic anticoagulation was prioritized.

Day 3 laboratory values were obtained 17 h after the Day 2 FFP and antithrombin administration, during ongoing UFH and before the additional FFP. Fibrinogen was 105 mg/dL, FDP 12.8 μg/mL, D-dimer 3.7 μg/mL, and antithrombin activity 80.6%. After decongestion, serum creatinine had decreased to 0.86 mg/dL (reference range, 0.46–0.79 mg/dL) and body weight to 36.3 kg by Day 39; thus, the prescribed pre-admission edoxaban dose of 30 mg once daily was consistent with the low-body-weight dose-reduction criterion. On Day 36, follow-up transthoracic echocardiography with an apical five- to four-chamber sweep no longer visualized the previously observed left atrial masses (see Supplementary material online, *Video S3*). CT on Day 39 showed complete disappearance of the filling defects (*Figure 1C* and *D*). No systemic embolism occurred. After stabilization, warfarin therapy was reintroduced with adherence support.

## Discussion

This case shows that surgical appendage resection does not eliminate the risk of left atrial cavity thrombosis. Rheumatic atrial remodelling, long-standing persistent atrial fibrillation, marked atrial enlargement, and severely impaired forward flow probably produced profound stasis despite the absence of an abnormal transprosthetic gradient. The marked pulmonary oedema and pleural effusions raised the possibility that the large left atrial thrombi disturbed pulmonary venous drainage into the left atrium and thereby aggravated pulmonary congestion. However, direct obstruction of the pulmonary vein ostia was not demonstrated, and concurrent heart failure therapy precluded causal attribution. Surgical appendage exclusion is an adjunct to, not a replacement for, systemic anticoagulation.^1,2^ Previous reports have described left atrial thrombi after mitral valve replacement and after appendage-targeted procedures.^3–5^

The event should not be interpreted simply as edoxaban failure. The prescribed pre-admission edoxaban dose of 30 mg once daily was consistent with the low-body-weight dose-reduction criterion after decongestion, but actual exposure was uncertain because adherence appeared suboptimal. Evidence for direct oral anticoagulants after bioprosthetic mitral valve replacement is evolving, whereas vitamin K antagonists remain favoured in rheumatic atrial fibrillation.^6–10^ With DOAC therapy, missed doses may remain less apparent unless adherence is actively assessed. In this case, warfarin was reintroduced with adherence support. International normalized ratio (INR) monitoring with warfarin also provided repeated opportunities to assess adherence.

Consumptive coagulopathy was an additional clinically relevant feature. The pretreatment ISTH score was 6, compatible with overt DIC.^11^ Massive intracardiac thrombi may sustain coagulation and fibrinolytic activation, although causality cannot be established; a similar association has been reported.^12^ UFH depends on antithrombin-mediated inhibition of thrombin and factor Xa, so reduced antithrombin activity provided a pathophysiological rationale for supplementation.^13^ However, the Day 3 changes occurred during UFH and heart failure therapy and after the Day 2 FFP and antithrombin concentrate. FFP and antithrombin administration may have influenced fibrinogen and antithrombin values, and the contribution of each intervention cannot be distinguished.

Contrast-enhanced CT established the diagnosis and documented resolution, whereas non-contrast CT was non-diagnostic. Transthoracic, but not transoesophageal, echocardiography was performed, medication adherence could not be objectively verified, and a residual appendage stump could not be completely excluded. The septal thrombus was remote from the appendage region, suggesting the thrombotic burden was not confined to a potential stump. Nevertheless, the imaging pattern and disappearance during anticoagulation strongly supported the interpretation that the filling defects represented thrombi.

## Conclusion

Giant left atrial cavity thrombi can occur after surgical left atrial appendage resection when rheumatic atrial remodelling, marked left atrial enlargement, severe atrial stasis, and uncertain anticoagulant exposure coexist.

## Lead author biography


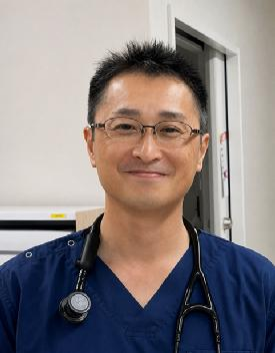


Takashi Akima, MD, PhD, is an internist and cardiologist at Saitama City Hospital, Japan. His clinical interests include atrial fibrillation, thrombosis, heart failure, and general internal medicine. He is involved in acute and complex inpatient care, clinical education, and case-based research. He has a particular interest in integrating cardiovascular expertise with broad-based internal medicine and translating clinically important observations into practical lessons for patient care.

## Supplementary Material

ytag778_Supplementary_Data

## Data Availability

The data underlying this case report are available within the article. Additional de-identified information may be available from the corresponding author upon reasonable request, subject to patient privacy considerations and institutional approval.
